# Reconsidering the interpretation of sperm DNA fragmentation testing in ART decision-making

**DOI:** 10.1093/hropen/hoag045

**Published:** 2026-05-19

**Authors:** Sandro C Esteves, Peter Humaidan

**Affiliations:** ANDROFERT, Andrology and Human Reproduction Clinic, Campinas, Brazil; Department of Surgery (Division of Urology), Faculty of Medical Sciences, University of Campinas (UNICAMP), Campinas, Brazil; Department of Clinical Medicine, Aarhus University, Aarhus, Denmark; Department of Clinical Medicine, Aarhus University, Aarhus, Denmark; University Clinic for Fertility, Skive Regional Hospital, Skive, Denmark

Dear Editor,

We read with interest the secondary analysis by Wang *et al*. (2026) in *Human Reproduction Open* evaluating whether sperm DNA fragmentation (SDF) testing can guide the choice between conventional IVF and ICSI in couples with non-severe male infertility. The authors concluded that testing has limited value for identifying couples who would benefit from ICSI over IVF. While the study provides valuable data derived from a large randomized trial, several methodological and statistical considerations suggest that this conclusion should be interpreted with caution.

First, the analysis includes only a subset of participants from the original trial (953 of 2329 couples) in whom SDF testing was available. As acknowledged by Wang *et al*. (2026), SDF testing was not routinely performed across all participating centers, thereby breaking the original randomization and converting this analysis into a nested observational cohort. Although adjustment for center effects was applied, statistical adjustment cannot fully account for selection mechanisms influencing which participants underwent SDF testing. Conditioning the analysis on the availability of SDF measurements may therefore introduce selection or collider bias, limiting causal interpretation.

Second, the study by Wang *et al*. (2026) appears underpowered to detect clinically meaningful modification of treatment effect by SDF. The reported median SDF of ∼18% suggests that most participants had non-pathological levels, whereas individuals with markedly elevated SDF—those most likely to benefit from ICSI—were relatively underrepresented. In the highest SDF quartile (≥25.9%), fewer than 250 couples were analyzed; this group likely contains substantial heterogeneity, combining individuals with moderately elevated fragmentation (i.e. 26–30%) with those exhibiting very high values (>35–40%). Categorizing SDF into quartiles rather than biologically relevant thresholds may therefore obscure effects in patients with very high SDF levels (e.g. >40%). Notably, the confidence interval for cumulative live birth in this subgroup (adjusted odds ratio 0.80; 95% CI 0.47–1.37) is wide and compatible with both clinically meaningful benefit and harm. Accordingly, the absence of statistical significance should not be interpreted as evidence that SDF does not influence the relative effectiveness of IVF and ICSI.

Third, the primary inference by Wang *et al*. (2026) relies on statistical tests for interaction between SDF and treatment effect. Such analyses require substantially larger sample sizes than main-effect analyses, particularly for continuous biomarkers (Freidlin *et al*. 2014). Thus, a non-significant interaction test does not exclude clinically relevant heterogeneity of treatment effect.

Interestingly, the only statistically significant signal observed by Wang *et al*. (2026) concerned total fertilization failure (TFF). In the highest SDF quartile, ICSI was associated with a lower risk of TFF compared with IVF (1.6% versus 7.0%). Although event numbers were small, this finding is biologically plausible and consistent with the ability of ICSI to bypass sperm–oocyte interaction defects associated with increased DNA damage (Esteves *et al*. 2021).

Importantly, emerging evidence indicates that not all forms of sperm DNA damage carry the same biological significance. In a recent study from our group using the neutral comet assay, increased double-strand SDF was associated with poorer reproductive outcomes in conventional IVF (Humaidan *et al*., 2026). These findings suggest that specific types of sperm DNA damage may differentially affect fertilization strategies and may not be fully captured by global SDF measurements alone, as used in the study of Wang *et al*. (2026).

These considerations are particularly relevant when interpreted alongside the original randomized trial comparing IVF and ICSI in couples with non-severe male factor infertility (Wang *et al*., 2024). We have previously noted that defining ‘non-severe male factor infertility’ solely on conventional semen parameters may overlook key aspects of sperm functional integrity, including DNA damage (Esteves and Humaidan 2024).

From this perspective, SDF testing may be most informative not as a predictor of overall ART success but as a tool for treatment stratification (Esteves and Humaidan, 2026). Directing patients with markedly elevated SDF towards ICSI while allowing those with lower fragmentation to undergo conventional IVF could mitigate the detrimental effects of sperm DNA damage on fertilization and early embryogenesis.

Finally, SDF should be considered within a broader clinical framework. Elevated SDF often reflects underlying male reproductive or systemic conditions—including oxidative stress, varicocele, metabolic disorders, and environmental exposures (Esteves and Humaidan, 2026). Identifying such abnormalities may enable targeted interventions aimed at improving paternal reproductive health and potentially influencing offspring health.

In conclusion, the study by Wang *et al*. (2026) contributes important data to an ongoing debate regarding the role of SDF testing in assisted reproduction. However, the methodological constraints of this secondary analysis and the limited power within high-SDF subgroups suggest that the findings may not yet provide a definitive answer regarding its clinical utility. Further prospective studies designed to evaluate biomarker-guided treatment strategies and the relevance of different types of sperm DNA damage may help clarify the potential role of SDF testing in optimizing ART management and outcomes.

## References

[hoag045-B1] Esteves SC, Zini A, Coward RM, Evenson DP, Gosálvez J, Lewis SEM, Sharma R, Humaidan P. Sperm DNA fragmentation testing: summary evidence and clinical practice recommendations. Andrologia. 2021;53:e13874. 33108829 10.1111/and.13874 PMC7988559

[hoag045-B2] Esteves SC, Humaidan P. Conventional in-vitro fertilisation versus intracytoplasmic sperm injection for male infertility. Lancet 2024;403:880–881. 38330979 10.1016/S0140-6736(23)02583-7

[hoag045-B3] Esteves SC, Humaidan P. Sperm DNA fragmentation: how to test, when to test, and what to do with abnormal results—a pragmatic mini-review for clinical practice. Hum Reprod 2026;deag056. 10.1093/humrep/deag056. Epub ahead of print. PMC13334924 42049202

[hoag045-B4] Freidlin B, Korn EL, Gray R. Marker Sequential Test (MaST) design. Clin Trials 2014;11:19–27. 24085774 10.1177/1740774513503739 PMC11406163

[hoag045-B5] Humaidan P, Povlsen BB, Drakeley AJ, Jensen MB, Gabrielsen AV, McDowell SH, Moore L, Ledgerwood CJ, Rae L, Sloan A et al Double-stranded sperm DNA fragmentation measured with neutral comet assay as a predictor of IVF outcomes: evidence from three European clinics in a multicentred prospective study. Hum Reprod 2026;41:677–688. 41903517 10.1093/humrep/deag046 PMC13139651

[hoag045-B6] Wang Y, Wang R, Lian Y, Yang R, Gao J, Liu J, Tang L, Liang X, Cao Y, Li W, et al Evaluating the potential for sperm DNA fragmentation testing to guide the use of ICSI for couples with nonsevere male infertility. Hum Reprod Open 2026; 2026: hoag011. 41836350 10.1093/hropen/hoag011 PMC12981913

[hoag045-B7] Wang Y, Li R, Yang R, Zheng D, Zeng L, Lian Y, Zhu Y, Zhao J, Liang X, Li W et al Intracytoplasmic sperm injection versus conventional in-vitro fertilisation for couples with infertility with non-severe male factor: a multicentre, open-label, randomised controlled trial. Lancet. 2024;403:924–934. 38330980 10.1016/S0140-6736(23)02416-9

